# Heat and Cold

**Published:** 1876-01

**Authors:** 


### HEAT AND COLD.

Our friend Dr. Mattson, whose beautiful Family Syringes have so long been a household necessity, here and in Europe, is investigating the subject of the application of heat and cold to the human body, in the treatment of diseased and painful conditions, and has reached some very remarkable results, which he will shortly give to the world in a suitable volume. The subject is certainly a most important one; and no gentleman of our acquaintance is better fitted by education and experience to present its features to the profession and the world. As to the value of cold in certain diseased states, we are prepared to testify in strong terms. Dr. Mattson was the first to show us and the world how instantly the terrible suffering attendant upon piles or hemorrhoids could be stopped by the use of the pile-cone used by him for the local application of dry cold. The effect is like magic in its suddenness, and has the added merit of being lasting. Most druggists have this cone, we think; and not one in the world ought to be without an ample supply.

---

DIARIES.—Diaries are very convenient affairs; but, as ordinarily arranged, they die out with the year, and unless used when fresh, are worthless. Now, we have a patented diary which holds good for five years, and is indeed a charmingly convenient affair, especially for physicians. It is published by the Erie Publishing Company, Erie, Pa., to whom orders should be addressed. The price, bound in red morocco, is $2.

